# Embryonic Lethality in Mice Lacking the Nuclear Factor of Activated T Cells 5 Protein Due to Impaired Cardiac Development and Function

**DOI:** 10.1371/journal.pone.0019186

**Published:** 2011-07-12

**Authors:** Man Chi Mak, Ka Man Lam, Ping Kei Chan, Yu Bond Lau, Wai Ho Tang, Patrick Ka Kit Yeung, Ben Chi Bun Ko, Stephen Man Sum Chung, Sookja Kim Chung

**Affiliations:** 1 Department of Anatomy, Li Ka Shing Faculty of Medicine, The University of Hong Kong, Hong Kong SAR, China; 2 Division of Science and Technology, United International College, Zhuhai, Guandong, China; 3 Department of Anatomical and Cellular Pathology, The Chinese University of Hong Kong, Hong Kong SAR, China; Children's Hospital Los Angeles, United States of America

## Abstract

Nuclear factor of activated T cells 5 protein (NFAT5) is thought to be important for cellular adaptation to osmotic stress by regulating the transcription of genes responsible for the synthesis or transport of organic osmolytes. It is also thought to play a role in immune function, myogenesis and cancer invasion. To better understand the function of NFAT5, we developed NFAT5 gene knockout mice. Homozygous NFAT5 null (NFAT5^−/−^) mouse embryos failed to develop normally and died after 14.5 days of embryonic development (E14.5). The embryos showed peripheral edema, and abnormal heart development as indicated by thinner ventricular wall and reduced cell density at the compact and trabecular areas of myocardium. This is associated with reduced level of proliferating cell nuclear antigen and increased caspase-3 in these tissues. Cardiomyocytes from E14.5 NFAT5^−/−^ embryos showed a significant reduction of beating rate and abnormal Ca^2+^ signaling profile as a consequence of reduced sarco(endo)plasmic reticulum Ca^2+^-ATPase (SERCA) and ryanodine receptor (RyR) expressions. Expression of NFAT5 target genes, such as HSP 70 and SMIT were reduced in NFAT5^−/−^ cardiomyocytes. Our findings demonstrated an essential role of NFAT5 in cardiac development and Ca^2+^ signaling. Cardiac failure is most likely responsible for the peripheral edema and death of NFAT5^−/−^ embryos at E14.5 days.

## Introduction

Nuclear factor of activated T cells 5 protein (NFAT5), also called tonicity element binding protein (TonEBP) [1] or osmotic response element binding protein (OREBP) [2], is a member of the Rel family of transcription factor with a conserved DNA binding Rel domain [3]. Although NFAT5 has similar a DNA binding domain as other NFATs (NFAT1–4), which are regulated by calcium/calcineurin and primarily involved in the regulation of cytokine and other genes important for the immune response in T lymphocytes [4], its regulation and biological functions are quite different from the other NFATs. When there is hypertonic stress, NFAT5 is translocated to the nucleus and regulates gene transcriptions, which are responsible for the import or synthesis of organic osmolytes such as myo-inositol, betaine, taurine, and sorbitol [5]. NFAT5 mRNA is also stabilized by hypertonic stress, leading to increased synthesis of NFAT5 [6]. Activated NFAT5 regulates the transcription of sodium/myo-inositol cotransporter (SMIT), sodium-chloride-betaine cotransporter (BGT1), and taurine transporter (TauT), which are responsible for the cellular uptake of myo-inositol, betaine and taurine, respectively. Transcription of aldose reductase (AR), which is involved in the synthesis of sorbitol, is also regulated by NFAT5 [7]. Apart from the osmoprotective genes, heat shock protein 70 (HSP70) gene also contains an osmotic response element (ORE), and its expression is regulated by NFAT5 under hypertonic stress [8]. The critical role of NFAT5 in osmoprotection has been demonstrated in NFAT5 knockout mice [9], [10]. The vast majority of the NFAT5 null mice died at the embryonic stage, and the few that survived to adult stage, exhibited kidney atrophy in the medulla with reduced level of AR, BGT1, and SMIT [10].

Besides the renal medulla, where the epithelial cells are constantly exposed to hypertonicity, NFAT5 mRNA has also been detected in the brain, heart and T lymphocytes [11], [12], suggesting it may have functions other than osmoprotection. In T lymphocytes, NFAT5 can be induced by both hypertonicity and mitogen [12]. NFAT5 is detected in some transformed cells which are integrin-mediated in carcinoma metastasis [13]. Moreover, several studies have suggested that NFAT5 plays a role in cell differentiation [10], [14], [15]. To better understand the physiological functions of this protein, NFAT5 knockout mice were generated and used in the present study. Here we show that the embryonic lethality for NFAT5 null mutant NFAT5^−/−^ mice is likely due to impaired cardiac development.

## Results

### NFAT5 Deficiency Caused Embryonic Lethality

**Figure 1:**
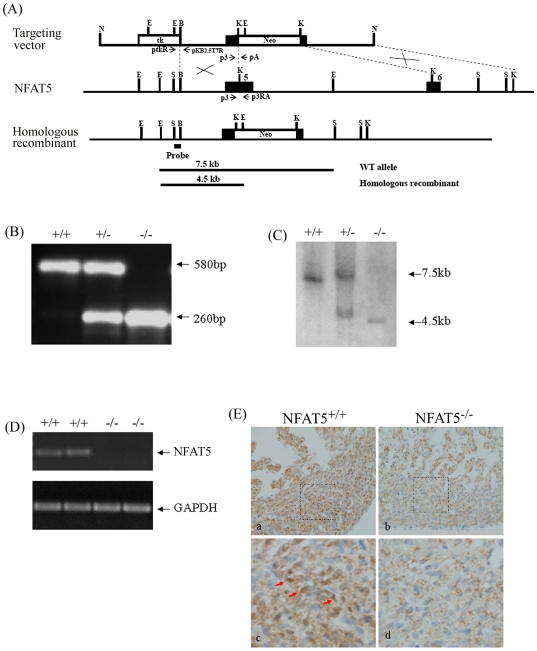
Targeting vector to generate NFAT5 null mutant mice. (A) The genomic structure of mouse *NFAT5* gene. Exons are represented in black boxes and numbered with 1–16. Introns are represented by lines. Targeting construct for NFAT5 knockout which contain the thymidine kinase gene (tk) and the neomycin resistance gene (Neo). The targeting vector was linearized with Not1 restriction endonuclease. Exon 5 and 6 are shown in boxes and the restriction map of the endogenous NFAT5 and the mutant allele after homologous recombination are shown. The primers (ptkR, pKB2.5T7R, p3.pA and p3RA) used for PCR screening are shown as arrows. For Southern hybridization screening, 600-bp Spe1-BamH1 fragment was used as external probe, the expected sizes of EcoRV fragments from wild type allele and homologous recombinant were 7.5 kb and 4.5 kb respectively. E: EcoRV, B: BamH1, K:Kpn1, N:Not1, S:Spe1 (B) Deletion of NFAT5 gene was verified by genotyping using PCR and (C) Southern blot analyses. (D) RT-PCR analysis of NFAT5 RNA in hearts from E14.5 wild-type and NFAT5^−/−^ embryos. (E) Representative photomicrography showing expression of NFAT5 in ventricular compact zone in E14.5 NFAT5^+/+^ embryonic hearts (red arrows), n = 5.

To define the physiological functions of NFAT5 we developed mice with null mutation in this gene. Homologous recombination between the target vector and the genomic DNA resulted in replacing part of exon 5 and exon 6 of NFAT5 gene by PGKNeo gene (Figure 1A). Deletion of these exons eliminates part of the nuclear localization signal and DNA binding domain of NFAT5. Heterozygous NFAT5 null (NFAT5^+/−^) mice appeared normal. Mating between NFAT5^+/−^ mice did not yield homozygous (NFAT5^−/−^) offspring while mating the NFAT5^+/+^ with NFAT5^+/−^ genotypes gave birth to the offspring following the Mendelian ratio, suggesting that NFAT5 null mutation was embryonic lethal, similar to that reported for NFAT5 null mice in another study [9]. To determine the onset of embryonic lethality, the genotypes of embryos of NFAT5^+/−^ matings were determined at different stages of development. As shown in Table 1, at E14.5 the ratio of NFAT5^+/+^, NFAT5^+/−^ and NFAT5^−/−^ genotypes showed no deviation from Mendelian transmission. However, from E17.5 onwards, NFAT5^−/−^ genotype was underrepresented, suggesting reabsorption of mal-developed NFAT5 null embryos in the uterus. No NFAT5 mRNA or protein was detected in the heart of E14.5 NFAT5^−/−^ embryos (Figure 1D, 1E), confirming NFAT5 deficiency in these mice.

**Table 1:** Embryonic lethality of NFAT5^−/−^ mice.

Day	NFAT5^**+**/**+**^	NFAT5^**+**/**−**^	NFAT5^**−**/**−**^
**E14.5**	29(29)	75(58)	35(29)
**E17.5**	14(14)	27(28)	0(14)
**P18–21**	70(70)	139(140)	3(70)

The numbers of mice of the indicated genotypes obtained from heterozygous intercrosses. Numbers in parentheses indicate the expected number based on predicted Mendelian inheritance.

### Cardiac Abnormalities in NFAT5^−/−^ Embryos

**Figure 2:**
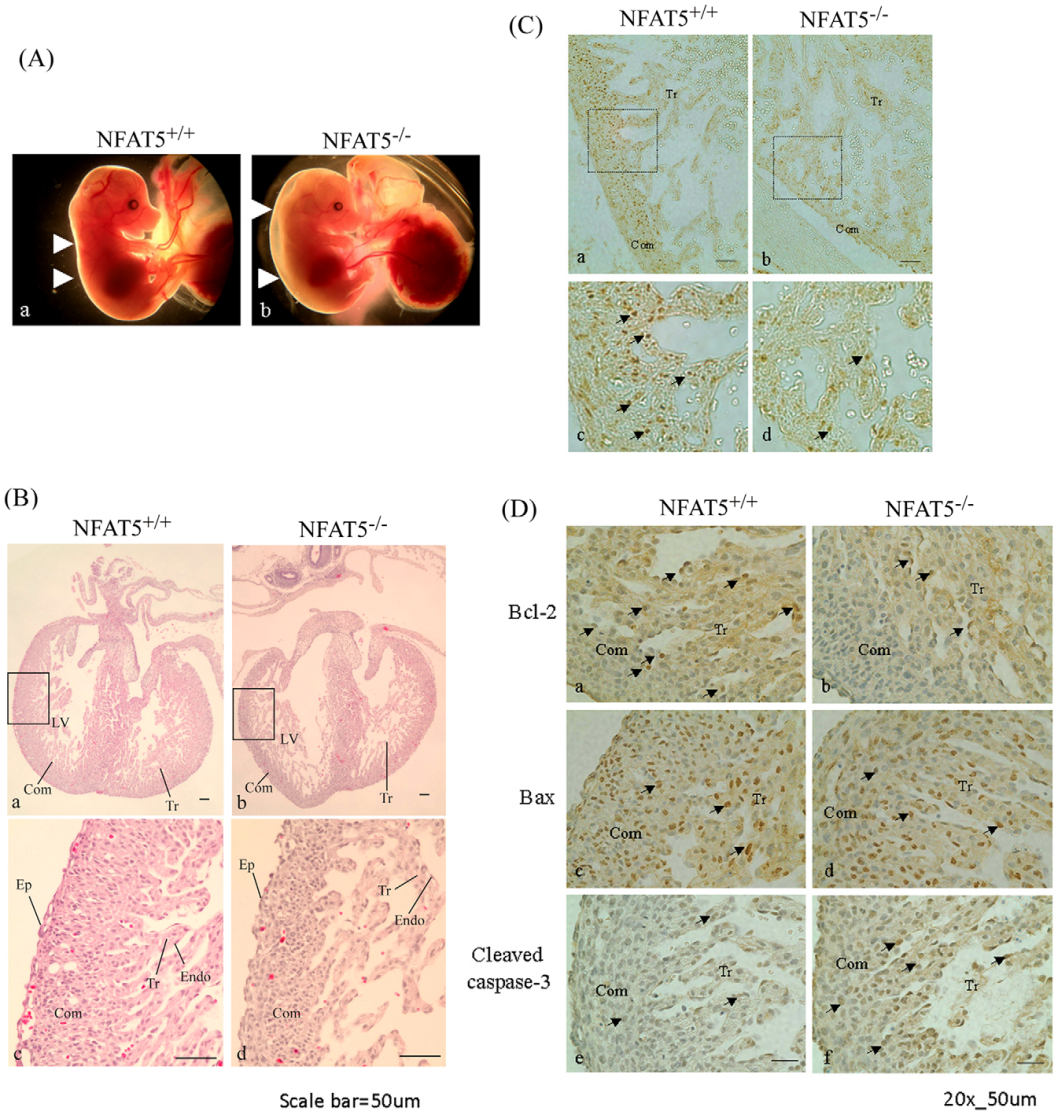
Peripheral edema in embryos at E14.5d. (A) Edema is apparent in the E14.5 NFAT5^−/−^ embryos (b) compared with a wild-type littermate (a). The arrow indicate the outer skin layer. (B) Histological comparison of NFAT5^+/+^ (a and c) and NFAT5^−/−^ (b and d) embryonic heart at 14.5 days. (C) Representative photomicrograph showing proliferating cell nuclear antigen (PCNA)-positive cells (arrows) in compact zone and trabecular region in E14.5 NFAT5^+/+^ and NFAT5^−/−^ hearts. (D) Representative photomicrography showing the (a and b) Bcl-2, (c and d) Bax and (e and f) cleaved caspase-3 staining (arrows) on E14.4 NFAT5^+/+^and NFAT5^−/−^ hearts. Com, compact layer; E, endocardial cushion; Endo, endocardium; Ep, epicardium; LV, left ventricle; Tr, trabecular. n = 5 Scale bar = 50 µm.

There was no gross structural abnormality observed in the E14.5 NFAT5^−/−^ embryos. These embryos exhibited spontaneous movements and cardiac pulsations. However, they appeared anaemic and oedematous, suggesting that these mice might suffer from heart failure (Figure 2A). The hearts of E14.5 NFAT5^−/−^ and NFAT5^+/+^ mice were sectioned and examined under microscope. The ventricular myocardium, septum and trabeculae of the wildtype embryos showed tissues with tightly packed cells organization (Figure 2B a and c). On the other hand, the hearts of NFAT5 null embryos showed significantly thinner ventricular wall and loosely packed cells organization in the compact and trabecular zone (Figure 2B b and d).

### Abnormal Cardiomyocyte Proliferation and Apoptosis in NFAT5^−/−^ Embryos

To further understand the structural abnormalities of the hearts of the NFAT5^−/−^ mice, sections of hearts from the E14.5 embryos were stained with antibodies against the proliferating cell nuclear antigen (PCNA), a marker for proliferating cells. The number of PCNA-positive staining cells in the compact and trabecular zone was significantly reduced in NFAT5^−/−^ embryos compared with NFAT5^+/+^ embryos (Figure 2C), suggesting less proliferating cardiomyocyte in NFAT5 null embryos. Bcl-2, Bax and cleaved caspase-3 staining was also preformed to determine whether abnormal regulation of apoptosis also contributed to the thinner ventricular wall and loosely packed cells organization in the trabeculae in the NFAT5 null embryos' hearts. The Bcl-2 positive cells were found in the compact zone and trabeculae of the NFAT5^+/+^ hearts (Figure 2D a), but rarely seen in that of NFAT5^−/−^ hearts (Figure 2D b). Bax positive cells were also found in the compact zone and trabeculae of the hearts, however, there was no significant difference between the NFAT5^+/+^ and NFAT5^−/−^ hearts (Figure 2D c and d). On the other hand, the number of cleaved caspase-3 positive cells was increased significantly in the compact zone, trabeculae and epicardium of the NFAT5^−/−^ embryos when compared with that of the NFAT5^+/+^ embryos (Figure 2D e and f).

### Reduced Beating Rate in NFAT5^−/−^ Embryonic Cardiomyocytes

**Figure 3:**
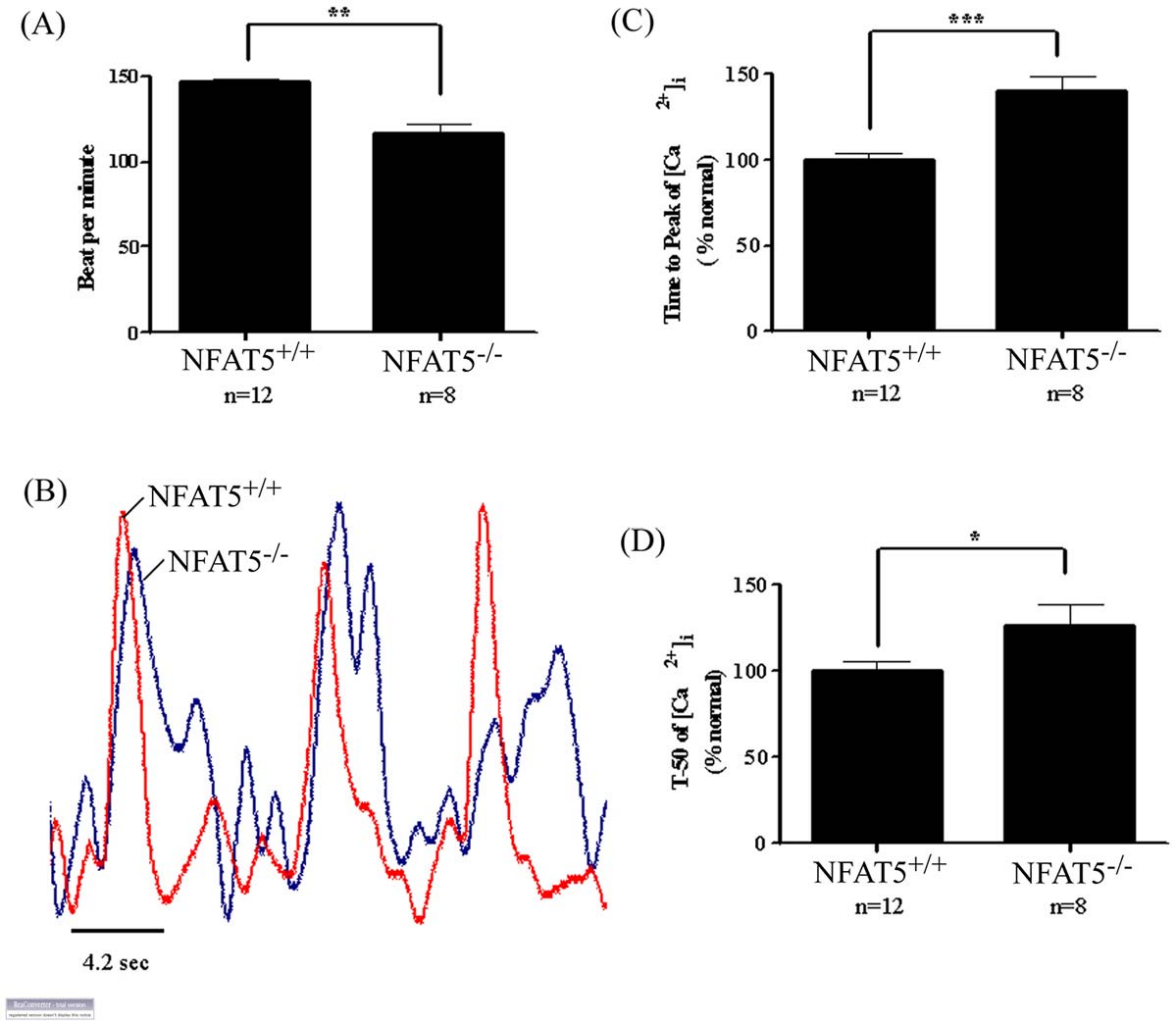
Beating rate of cardiomyocytes from E14.5 embryos. (A) The beating rate is presented in total counts of beating in cardiomyocytes per minute. Data presented as mean ± S.E.M. **P<0.01, one-way ANOVA. (B) Ca^2+^ records from a rested (not paced) NFAT5^+/+^ and NFAT5^−/−^ cardiomyocytes. Effect of NFAT5 on amplitude of [Ca^2+^]i transient (C), time to peak (D) and time to 50% decay (t_50_) in single ventricular cardiomyocytes. Data presented as mean ± S.E.M. ≥13 cardiomyocytes were measured from each individual animal. NFAT5^+/+^, n = 6; NFAT5^−/−^, n = 5 *P<0.05; ***P<0.0001, student t-test.

To determine if deficient of NFAT5 would affect the function of the cardiomyocytes, cardiomyocytes were isolated from the hearts of E14.5 NFAT5^+/+^ and NFAT5^−/−^ embryos. Cardiomyocyte were cultured for 4 days and the beating rate of the synchronized cells was measured. The number of beats per minute in NFAT5^−/−^ cardiomyocytes was reduced significantly compared with that of the NFAT5^+/+^ cardiomyocytes (Figure 3A).

### Effect of NFAT5 Deficiency on Intracellular Calcium Signaling

To determine if the reduced beating rate of the NFAT5^−/−^ cardiomyocytes was due to the abnormal Ca^2+^ signaling, intracellular Ca^2+^ pulses, [Ca^2+^]i, in NFAT5^−/−^ and NFAT5^+/+^ cardiomyocytes were monitored using Ca^2+^ indicator, fura-2. When compared with the NFAT5^+/+^ cardiomyocytes, the Ca^2+^ oscillation profile of the NFAT5^−/−^ cardiomyocytes appeared oscillating irregularly (Figure 3B). In line with reduced beating rate results, the peak-to-peak time of Ca^2+^ wave in NFAT5^−/−^ cardiomyocytes was increased. The amount of Ca^2+^ in the cytoplasm, represented by the amplitude of [Ca^2+^]i, was similar between NFAT5^−/−^ and NFAT5^+/+^ cardiomyocytes. The rate of Ca^2+^ released from the sarcoplasmic reticulum (SR), represented by the time to peak of [Ca^2+^]I, was increased by 40% in NFAT5^−/−^ cardiomyocytes compared with that of wildtype cells (Figure 3C). The removal of cytoplasmic Ca^2+^, represented by the decay of [Ca^2+^]i, was also increased in NFAT5^−/−^ cardiomyocytes. The t_50_ of [Ca^2+^]i decay was increased to 126% in the NFAT5^+/+^ cardiomyocytes (Figure 3D).

### Effect of NFAT5 Deficiency on the Expression of RyR and SERCA in Ventricular Cardiomyocytes

**Figure 4:**
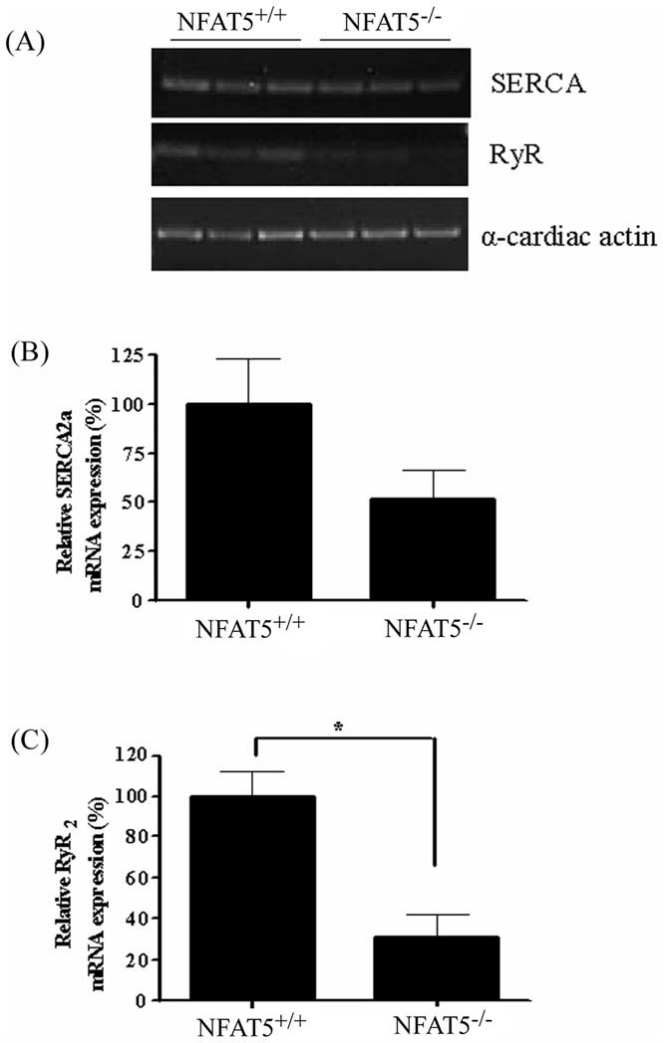
Effect of NFAT5 on the mRNA expression of RyR and SERCA in embryonic cardiomyocytes. (A) Representative semi-quantitative RT-PCR showing the relative mRNA abundance of SERCA and RyR in NFAT5^−/−^ cardiomyocytes compared to wild-type control. (B) Histogram showing the relative quantification of SERCA of semi-quantitative RT-PCR. (C) Histogram showing the relative quantification of RyR of semi-quantitative RT-PCR. Data presented as mean ± S.E.M. **P<0.05, student t-test, n = 3.

Since RyR and SERCA are the major proteins involved in Ca^2+^ release and uptake into SR, respectively. The mRNA levels of these two proteins in NFAT5^+/+^ and NFAT5^−/−^ cardiomyocytes were determined by semi quantitative RT-PCR (Figure 4A). There was a trend of reduction in SERCA mRNA level in NFAT5^−/−^ cardiomyocytes, however, the difference was not statistically significant (Figure 4B). The RyR mRNA level, on the other hand, was significantly reduced in NFAT5^−/−^ cardiomyocytes (Figure 4C). The reduced expression of these proteins is likely to contribute to contractile dysfunction in NFAT5^−/−^ cardiomyocytes.

### Downregulation of HSP 70 and SMIT mRNA Expression in NFAT5^−/−^ Cardiomyocytes

NFAT5 is a key factor that enhances the transcription of AR, SMIT, TauT and HSP70 when cells are under hypertonic stress. To determine if NFAT5 deficiency affects the expression of these genes under isotonic condition, the mRNA levels of these genes in NFAT5^−/−^ and NFAT5^+/+^ cardiomyocytes were determined by real-time RT-PCR. The levels of AR and TauT mRNA were decrease in NFAT5^−/−^ cells, but the difference was not statistically significant. HSP 70 and SMIT mRNA levels were reduced significantly in NFAT5^−/−^ cardiomyocytes (Table 2), indicating that even under isotonic condition, NFAT5 is involved in the transcription of these genes.

**Table 2:** Real-time PCR analysis of genes downstream to NFAT5 in embryonic cardiomyocytes.

mRNA	NFAT5^**+**/**+**^	NFAT5^**−**/**−**^
**AR**	1.23±0.53	0.17±0.08
**HSP70**	1.00±0.04	0.30±0.10**
**SMIT**	1.00±0.02	0.30±0.12**
**TauT**	1.11±0.35	0.68±0.04

Values are means ± S.E.M.

**P<0.01 vs NFAT5^+/+^ control, student t-test, n = 3.

## Discussion

A previous study has shown that NFAT5 null mice with a deletion of the sixth exon in the NFAT5 allele, which encodes the DNA-binding loop of NFAT5, shows kidney abnormalities [9]. Go et al., 2004 generated other NFAT5 mutant mice with exons 6 and 7 deletions, which eliminated a region of the NFAT5 protein important for the site-specific DNA-binding transcription factor. Immune function impairment is shown in these mice. In the present study, we have generated the NFAT5 null mice with partial exon 5 and 6 deletions. Similar reports for NFAT5 deficient mice [9], [10], we found that NFAT5 deficiency is embryonic lethal. A reduction in the number of NFAT5^−/−^ embryos after E14.5 days suggests that these embryos died soon after E14.5 and were reabsorbed in the uterus. In the previous studies, only adult mice were used to investigate the role of NFAT5, and the importance of embryonic lethality was not addressed. The kidney defects reported in newborn NFAT5 deficient mice were not the cause of embryonic lethality because the kidney was not yet functioning at that stage [9]. In the present study, we show that the most prominent feature of the E14.5 NFAT5^−/−^ embryos is peripheral edema. Increased permeability of the blood vasculature might contribute to this edema. Animal models with increased vascular permeability showed signs of edema with extensive hemorrhage [16], [17]. However, this may not be the mechanism that caused edema in NFAT5^−/−^ embryos as they did not exhibit any obvious hemorrhage. Edema and mid-gestation lethality are signs of congestive cardiac failure, which results in back-pressure that extrudes circulation fluid into the tissues [18]. To investigate if this was the cause of edema in NFAT5^−/−^ embryos, the structure of the hearts and the function of cardiomyocytes was examined. The developed hearts of E14.5 NFAT5^−/−^ embryos appeared abnormal with a thinner ventricular wall and lower cell density at the compact and trabecular zone of myocardium. The functions of cardiomyocytes in these embryos also seemed impaired, as indicated by a slower beat rate. Taken together, these facts suggest that cardiac failure contributed to the edema and lethality for NFAT5^−/−^ embryos.

The exact mechanism by which NFAT5 affects cardiac development is still largely unknown. A recent study has reported that NFAT5^−/−^ T lymphocytes undergo cell cycle arrest in G1/S and G2/M, which is associated with the reduced expression of cyclins E1, A2 and B1 [19]. In the present study, PCNA-positive staining cells in the compact and trabecular zone were significantly reduced in NFAT5^−/−^ embryos compared with NFAT5^+/+^ embryos, suggesting that the cardiomyocytes were in a non-active proliferative stage, which is in agreement with the study indicating that NFAT5 deficiency causes cell cycle arrest. Together with the observed decrease in Bcl-2 and the increased cleaved caspase-3 expressions in cardiac tissues of NFAT5^−/−^ embryos, the thinner ventricular wall and reduced cell density in the cardiac tissues of NFAT5^−/−^ embryos is due to the increase in apoptosis and the decrease in cell proliferation [20]. Cardiomyocytes from NFAT5^−/−^ embryos exhibited a reduced spontaneous beat rate. This was associated with an abnormal Ca^2+^ signaling profile with increased time-to-peak and increased time-of-decay for the Ca^2+^ signal, resulting in a slower beat rate. This is probably due to decreased expressions of RyR and SERCA, as shown in the present study, which are the main proteins responsible for releasing Ca^2+^ from the SR to the sarcoplasm and for the uptaking of Ca^2+^ from the sarcoplasm to the SR, respectively.

Whether NFAT5 directly affect the expression of Bcl-2, PCNA, SERCA or RyR is not clear. NFAT5 is thought to be activated by hypertonicity. In the present study, however, when cardiomyocytes were cultured in isotonic condition, the expression levels of SMIT and HSP 70, which are known to be regulated by NFAT5 under hypertonic condition, were reduced in NFAT5^−/−^ cardiomyocytes, indicating that NFAT5 is also activated under isotonic condition. This is in agreement with the study indicating that NFAT5 expression is found in the nucleus under isotonic condition [21]. The speculation is that NFAT5 might also regulate the expression of Bcl-2, PCNA, SERCA and RyR during embryonic development.

Decreased expression of SMIT and HSP 70 might also contribute to the impaired function or development of the heart in NFAT5^−/−^ embryos. SMIT is responsible for importing myoinositol (MI) into the cells. MI is the precursor for the synthesis of inositol-3-phosphate (IP_3_), a signal transduction molecule involved in the regulation of many cellular functions, including the release of Ca^2+^ from the SR, and thus cardiac contraction [22], [23]. A reduced level of SMIT might lead to a lower IP_3_ level and impair cardiac contractility. HSP 70 is important for cell division during embryonic development [24] as it triggers the differentiation of the mesenchymal stem cells to form myocytes. Mice lacking HSP 70 show a mild cardiac hypertrophy and impaired cardiac contractile function [25]. It has been suggested that HSP 70 plays a role in Ca^2+^ signaling by interacting with Ca^2+^ handling proteins such as RyR, SERCA and NCX [26]. Thus, NFAT5 might also affect cardiac development and function by regulating the expression level of HSP 70.

Previous studies show that NFAT5 is important for kidney function [9] and T-cell development in the thymus [10], [27]. In the present study, we have demonstrated that NFAT5 also plays an important role in cardiac development and function. Embryonic lethality for NFAT5 deficient mice is most likely due to the impaired development and function of the heart.

## Materials and Methods

Mice were housed under diurnal lighting condition and allowed free access to food and water. The protocol of this study was reviewed and approved by the Committe on the Use of Live Animals in Teaching and Research in The University of Hong Kong.

### Generation of NFAT5^−/−^ Mice

The gene-targeting vector was constructed as shown in Figure 1A. It contains the thymidine kinase gene (tk), and a 2.5 kb of NFAT5 genomic DNA with part of exon 5 and 6 replaced by neomycin resistance gene (Neo). The NFAT5 targeting vector was transfected into AB2.2 embryonic stem (ES) cells by electroporation. ES cells with the targeting vector integrated into their genome by homologous recombination were selected by the addition of G418 and FIAU in the culture medium after transfection [28]. Two of the independent ES clones with the targeting vector integrated into the genome were injected into blastocytes from C57BL/6J mice. The blastocytes were implanted into the uterus of ICR foster mothers to carry the embryos to term. Mice with deletion of part of exon 5 and 6 of NFAT5 gene were identified by PCR using the following primers, p3: 5′-AGGCACACAGTCTTGTACATCTCAC-3′; p3RA: 5′-CCTCTATGCCTAACCATACATAA-3′ and pA: 5′-gatcagcagcctctgttcca-3′ (Figure 1B and C). Chimeric mice derived from blastocyte injections of gene-targeted NFAT5^+/−^ ES cells were backcrossed to C57BL/6 mice once, and germline transmission was verified by using PCR and southern blot hybridization (Figure 1C).

To generate NFAT5^−/−^ embryos, timed heterozygous matings were set up, and the morning of vaginal plug detection was considered to be embryonic day 0.5 (E0.5 d). The genotypes of the embryos were identified by PCR amplification.

### Histological and Immunohistochemical Analyses

For histological studies, 7 µm sections of paraffin-embedded hearts from 14.5 days old embryos (E14.5) were fixed with 4% paraformaldehyde, and stained with hematoxylin and eosin (H&E). For immunohistochemical (IHC) analyses, embryonic heart sections were incubated with antibodies against Bax, Bcl-2, cleaved caspase-3 (1∶200; Cell signaling, St Louis, MO, USA), PCNA and NFAT5 (TonEBP, 1∶400, a generous gift from Prof. H.M. Kwon, University of Maryland). The presence of antibody binding was visualized by Vectastain ABC kit (Vector Laboratories, Burlingame, CA, USA) with 3,3′-diaminobenzidine tetrahydrochloride (Zymed, South San Francisco, CA, USA). Photomicrographs were taken with a Zeiss Axiophot microscope. For controls, adjacent sections were used but without primary antibodies.

### Primary Cultures of Embryonic Cardiomyocytes

Embryonic cardiomyocytes isolation was performed as described previously [29]. Hearts of E14.5 embryos were dissected and kept in ice-cold Moscona's solution (136.8 mM NaCl, 28.6 mM KCl, 11.9 mM NaHCO_3_, mM 9.4 glucose, and 0.08 mM NaH_2_PO_4_, pH7.4). Atria were removed from the ventricles under a dissecting microscope (Olympus). Isolated ventricles were rinsed with ice-cold Hank's balanced salt solution (HBSS). The ventricles were minced and incubated with HBSS with 100 U/ml collagenase (type II, Worthington) in a 37°C water bath for 1 hour. At 15-min intervals, the tissues were pipetted up and down to dissociate the cells. The process was repeated until all tissues were dissociated. Cells were pelleted, resuspended in culture medium (DMEM supplemented with 10% FBS, 100 U/ml streptomycin and 100 U/ml penicillin, pH 7.4). Cells were plated at the density of 200 cells/mm^2^ on cell culture dishes pre-coated with 50 µg/ml of type I collagen (Vitrogen) in DMEM for 1 hour at 37°C. Synchronized beating cardiomyocytes colonies were selected for beating rate count. The rate of beating was presented in numbers of beating/minute.

### Measurement of [Ca^2+^]_i_ Transients in the Single Ventricular Myocytes

[Ca^2+^]_i_ transients were measured using a spectrofluorometric method with fura-2 AM as the Ca^2+^ indicator. Ventricular myocytes were incubated with 5 µm fura-2 AM for 30 min. Fluorescent signals obtained at 340-nm with 380-nm excitation wavelengths were recorded and stored in the computer for data processing and analysis.

### RNA Isolation, cDNA Preparation and Real-time PCR for Measurement of Abundance of Specific RNAs

Total RNA was isolated from ventricular myocytes using a TaqMan gene expression cells-to-C_T_™ Kit (Applied Biosystems). cDNA was prepared with TaqMan reverse transcription reagents, using random hexamers, according to the manufacturer's instructions (Applied Biosystems). cDNA was quantitated with ABI Prism 7900HT sequence detection system (Applied Biosystems). The accumulation of the PCR product is monitored in real time by a fluorogenic 5′-nuclease assay, using probes specific for each cDNA being tested. Primers and probes were designed from mouse cDNA sequences. The PCR primers were designed to span an intron of genes that contains introns, namely SMIT (Mm00444330_s1, Applied Biosystems), TauT (Mm00436909_m1, Applied Biosystmes), 18S rRNA primers and 18S probes (Applied Biosystems), AR and HSP 70-2 [30].

### Real-time PCR Analysis

The results were analyzed using ABI Prism 7900 system software (Applied Biosystems). The ABI Prism 7900 system records the number of PCR cycles (Ct) required to produce an amount of product equal to constant threshold value, set to be reached during the exponential phase of the PCR reaction. Relative mRNA abundance was calculated from the real-time PCR data.
